# Chronic Kidney Disease is associated with an increase of Intimal Dendritic cells in a comparative autopsy study

**DOI:** 10.1186/s12950-015-0073-4

**Published:** 2015-03-29

**Authors:** Miguel Hueso, Joan Torras, Marta Carrera, August Vidal, Estanis Navarro, Josep Grinyó

**Affiliations:** Department of Nephrology, Hospital Universitari Bellvitge, and Institut d’Investigació Biomèdica de Bellvitge (IDIBELL), L’Hospitalet de Llobregat, C/ Feixa llarga, s/n; L’Hospitalet de Llobregat, 08907 Barcelona, Spain; Department of Pathology, Hospital Universitari Bellvitge, Barcelona, Spain; Laboratori d’Oncologia Molecular, Institut d’Investigació Biomèdica de Bellvitge (IDIBELL), L’Hospitalet de Llobregat, Spain

**Keywords:** Atherosclerosis, Chronic kidney disease, Inflammation, Nuclear factor-k B, Dendritic cells

## Abstract

**Background:**

Chronic Kidney Disease (CKD) and inflammation are risk factors for atherosclerotic vascular disease (ASVD). In inflammatory conditions, Nuclear Factor-κB (NF-κB) is frequently activated and it has been detected in human ASVD. In this work, we investigated if the degree of inflammation and of NF-κB activation were increased in the aorta of patients with CKD.

**Methods:**

This is a case–control pilot study performed on 30 abdominal aorta samples from 10 human autopsies. Cases were patients with CKD and controls patients with normal glomerular filtration rate (eGFR). Infiltrating mononuclear cells (S100^+^, CD3^+^, CD40^+^, CD40L^+^) and activation of NF-κB were identified by immunohistochemistry.

**Findings:**

The number of cells in the intima which showed activated nuclear NF-κB correlated with severity of ASVD lesions (r = 0.56, p = 0.003), with numbers of CD3^+^ lymphocytes in adventitia (r = 0.50, p = 0.008), with numbers of CD40^+^ cells in the intima (r = 0.59, p = 0.002) or in the adventitia (r = 0.45, p = 0.02), and with numbers of CD40L^+^ cells in the intima (r = 0.51, p = 0.011). Increased numbers of S100+ Intimal Dendritic cells (IDCs) were associated with ASVD (p = 0.03) and CKD (p = 0.01).

**Conclusions:**

Number of CD3^+^ cells, of CD40^+^ cells, of CD40L^+^ cells and the degree of NF-κB activation were increased in ASVD lesions suggesting a role for the adaptive T cell in the development of ASVD lesions. IDCs were associated both with ASVD and CKD suggesting a role of these cells in the pathogenesis of ASVD in CKD.

## Findings

### Atatement of the research

The higher incidence of atherosclerotic vascular disease (ASVD) in CKD could result from systemic and/or local vascular inflammation, in which activated T lymphocytes would play a critical role, since their deficiency in a mouse model has been shown to lead to a marked reduction of lesions, while their adoptive transfer reconstituted the disease [1]. Activation of T lymphocytes requires a second signal provided by costimulatory molecules expressed in the membrane of antigen presenting cells (APC). CD40 and its ligand CD40L (CD154) are critical components of the costimulatory pathway. CD40/CD40L signalling occurs via activation of NF-κB (Nuclear Factor-κB) which activates many of the genes involved in the inflammatory response that are pivotal in the pathogenesis of vascular injury [2]. Consequently, activated NF-κB has been detected in a rabbit model of ASVD [3] and in human ASVD but not in normal vessels [4]. Furthermore, activation of NF-κB has been also involved in the progression of renal tubulointerstitial lesions in experimental proteinuric nephropathies and in the development of human glomerulonephritis [5], a possible link between CKD and ASVD.

In this work we have studied the adaptive immune response in the aortic wall of patients with CKD, with the aim to explain the high incidence of ASVD in patients with CKD.

## Methods

### Study design

This is a case–control, pilot study using aortas from patients deceased in the Hospital Universitari de Bellvitge (HUB, 11/2009-02/2010). Cases were stage 3 (or higher) CKD patients at the time of hospital admission. Controls were patients, deceased in the same time-period, with a normal eGFR (estimated glomerular filtration by the “Modification of Diet in Renal Disease” simplified equation). The presence of CKD was confirmed by histological examination.

### Ethics statement

In this work, we have used authorized autopsy material from the Department of Pathology of the Hospital Universitari Bellvitge (HUB). Confidential information from patients was protected following national normatives. This manuscript has been revised by the Clinical Research Ethics Committee of the HUB.

### Immunohistochemistry

Three samples were isolated from each patient: macroscopically normal abdominal aorta, incipient ASVD lesion, and a complicated lesion. Sections were H/E-stained and classified according to the modified classification of American Heart Association (AHA) [6]. Formalin-fixed, paraffin-embedded aortas, were sliced at 4 μm and stained with polyclonal anti-CD3, anti-S100 (Dako, Glostrup, Denmark), anti-CD40, anti-CD40L (Santa Cruz Biotechnology, Santa Cruz, CA, USA), and anti-NF-κB-p65 (phosphor S536) (Abcam, Cambridge, UK) antibodies. Sections were counterstained with hematoxylin to make nuclei evident. Human parotid glands or ganglions were used as positive controls. Isotype controls were performed using antibody buffer supplemented with irrelevant immunoglobulines of the same isotype, species and concentration as the primary antibody (ThermoFisher, Rockford, IL USA). Negative controls were performed by omitting primary antibodies. Positively stained cells were counted at x100 to x200 and acquired with a digital camera. The percentage of positive cells was assessed irrespective of the staining intensity. Results were expressed as the percentage of each population regarding the total number of cells in the intima or adventitia.

### Statistical analysis

Cell infiltration was measured as the mean of scores ± standard deviation. Differences in cell infiltration between arterial lesions were compared by the Kruskal-Wallis test. Mann–Whitney test was used to compare differences in cell infiltration in CKD patients. Correlation among CD3, S100, CD40, CD40L and NF-κB scores in ASVD lesions against the presence of CKD was performed with the Spearman rank correlation coefficient. A p <0.05 value was considered statistically significant. P values were corrected for the number of variables compared according to the Bonferroni method. Statistical analysis was performed using the SPSS 12.0 software (SPSS Inc. Chicago, IL).

## Results and discussion

Atherosclerotic vascular disease (ASVD) is more common and more severe in CKD patients than in the general population [1,7] and ASVD lesions grow faster in an uremic environment [8] suggesting a pathogenic link. Since inflammation seems critical in the development of ASVD pathogenesis [9], the higher incidence of ASVD in CKD could result from systemic and/or local vascular inflammation. Thus, we present a pilot study in 30 samples from abdominal aortas, obtained from 10 deceased patients (Table 1) to investigate whether the abdominal aorta from CKD patients presented features of enhanced inflammation that would justify the increase in ASVD lesions observed in these patients.

**Table 1:** **Clinical characteristics of patients**

**ID**	**Age**	**Gender**	**Cause of death**	**Diabetes**	**Hypertension**	**Dyslipidemia**	**Creatinine (μmol/L)**	**eGFR (mL/min)**	**Histology**	**CKD stage**
1	**60**	**Female**	Cardiovascular	No	yes	No	Dialysis		FSGS + IF-TA	5D
2	**83**	**Female**	Cardiovascular	No	yes	No	66	>60	IF-TA	No
3	**83**	**Male**	Cancer	Yes	Yes	No	219	25	FSGS + IF-TA	4
4	**68**	**Female**	Cancer	No	No	No	83	>60	Normal	No
5	**49**	**Male**	Cardiovascular	No	Yes	Yes	327	17	FSGS + IF-TA	4
6	**60**	**Female**	Cardiovascular	Yes	Yes	Yes	84	>60	Normal	No
7	**68**	**Female**	Infection	No	Yes	No	130	35	IF-TA	3
8	**76**	**Male**	Infection	Yes	Yes	No	48	>60	multicystic	No
9	**86**	**Female**	Cardiovascular	No	Yes	No	169	21	FSGS + IF-TA	4
10	**85**	**Female**	Cardiovascular	Yes	Yes	No	66	>60	FSGS	No

Abbreviations:

ID: Identification Number.

eGFR: estimated Glomerular Filtration Rate.

CKD: Chronic Kidney Disease.

FSGS: focal segmental glomerulosclerosis.

IF-TA: Interticial Fibrosis and Tubular Atrophy.

**Figure 1:**
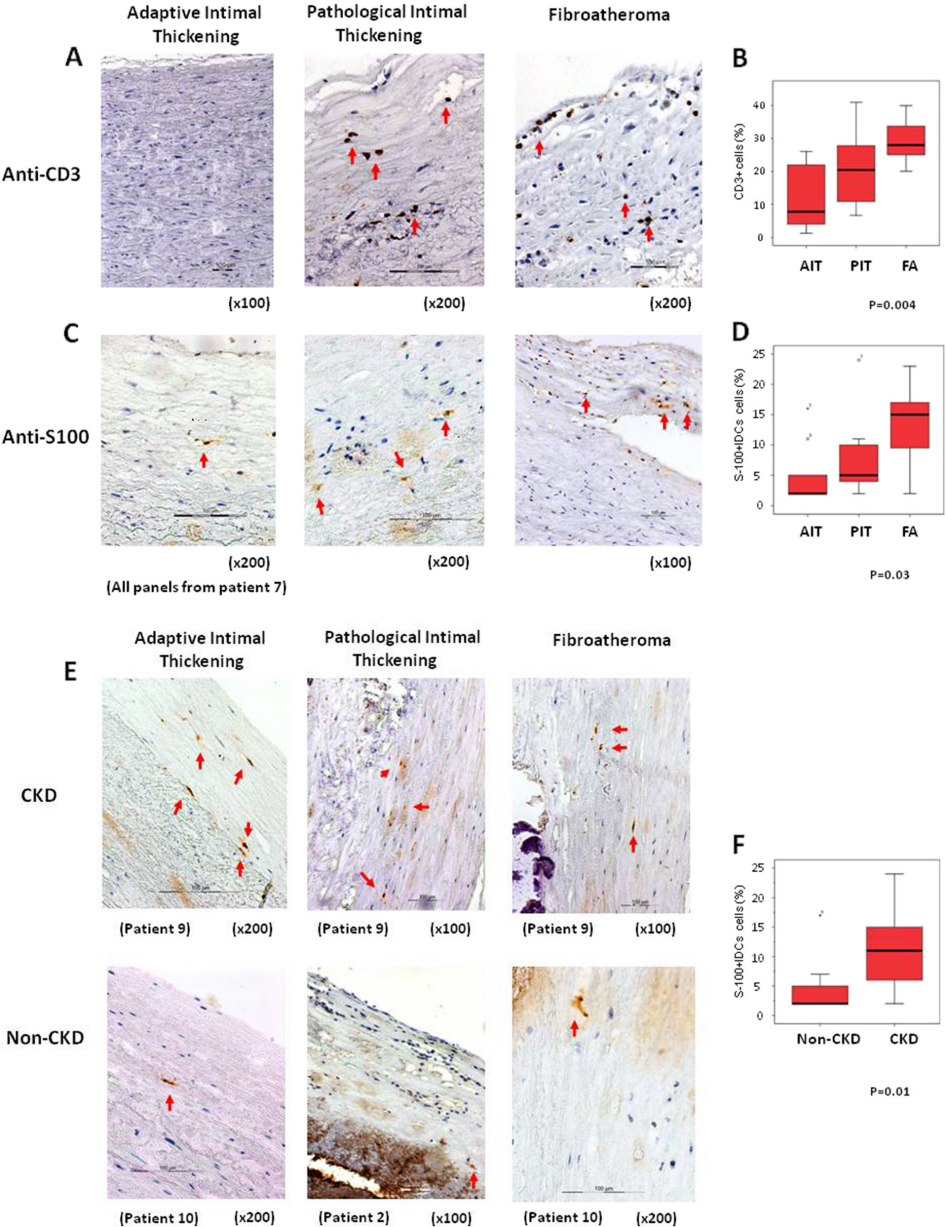
**Increased number of CD3+ T-lymphocytes and IDCs in areas of progressive ASVD lesions. A**. CD3+ T-lymphocytes detected in the endothelium and subendothelial zones of aortas with PIT or FA (arrows). Magnifications as indicated. **B**. Box-plot showing percentage of CD3+ T-lymphocytes, regarding the total number of cells in the field, according to histological lesion. Kruskal-Wallis test. **C**. S100^+^ IDCs, localized in the intima of aortas according to histological lesion. Kruskal-Wallis test. Magnifications as indicated. **D**. Box-plot showing percentage of S-100^+^IDCs according to histological lesion. Kruskal-Wallis test. **E**. S100^+^ IDCs, localized in the intima of aortas grouped according to histological lesions and presence of CKD. Magnifications as indicated. **F**. Box-plot showing percentage of S100^+^IDCs according to CKD and with independence of histological lesion. Mann–Whitney test. In figures B, D and F, each diagram represents the median, quartiles and outliers. The colored box represents the interquartile range that contains 50% of the values. The whiskers are lines that extended from the box to the highest and lowest values, excluding outliers. A line across the box represents the median value.

A few infiltrating CD3^+^ cells were found in the intima of aortas with adaptive intimal thickening (AIT) with their number increasing significantly in areas with pathological intimal thickening (PIT) or with fibroatheroma (FA) (Table 2), and correlating with the severity of the ASVD lesions (Figure 1A and B). These results are not surprising since inflammation seems to have a critical role in ASVD pathogenesis [9], subsequent to the development of maladaptive vascular inflammation as well as to the failure of inflammation-resolving regulatory processes in response to the deposition and modification of low-density lipoproteins within arterial walls [10].

**Table 2:** **Relative proportions of infiltrating cells in aorta samples from all patients classified according to the modified classification of AHA**

		**AIT**	**PIT**	**FA**	
**Mab**	**Location**	**n = 10**	**n = 10**	**n = 10**	**p**
αCD3	Intima	12 ± 9%	20 ± 9%	29 ± 6%^t^	0.005
	Adventitia	8 ± 6%	21 ± 23%	22 ± 14%	0.02
αS100	Intima	5 ± 5%	7 ± 7%	13 ± 6%	0.03
	Adventitia	11 ± 9%	9 ± 5%	9 ± 3%	0.97
αCD40	Intima	35 ± 20%	69 ± 17%^t^	73 ± 14%^t^	0.003
	Adventitia	59 ± 19%	77 ± 14%	71 ± 12%	0.05
αCD40L	Intima	30 ± 16%	59 ± 22%*	61 ± 25%*	0.01
	Adventitia	51 ± 15%	52 ± 20%	47 ± 13%	0.86
αNF-κB	Intima	14 ± 16%	45 ± 28%*	54 ± 25%^t^	0.01
	Adventitia	12 ± 9%	46 ± 35%*	40 ± 18%	0.01

For each case we calculated the number of CD3^+^, S100^+^, CD40^+^, CD40L^+^ and NF-κB^+^ infiltrating cells as percentage of total cells and expressed the result as the mean ± SD.

*P < 0.05 vs AIT (Bonferroni Test); ^t^P < 0.01 vs AIT (Bonferroni test)

Abbreviations:

AIT: adaptive intimal thickening,

PIT: pathological intimal thickening,

FA: fibroatheroma.

Since Dendritic Cells (DCs) are key sensors of the innate and the adaptive arms of the immune system, we analyzed their presence in the aortic wall. There are several markers for the identification of DCs but it has been suggested that S100 is the most reliable marker for the visualization of the major portion of DC population in the human arterial intima [11]. Although S100 is not exclusively specific to DC and it is expressed by neural cells, S100 specifically identifies DC in the arterial intima because the intima and two-thirds of the internal part of the tunica media are not innervated [12]. The proportion of S100^+^ Intimal Dendritic Cells (IDCs) was also increased in progressive ASVD lesions (Table 2 and Figure 1C) and in patients with CKD (11 ± 6% of IDCs in CKD aortas vs. 5 ± 5% in aortas without CKD, p = 0.01; Figure 1E and F), suggesting a role of these cells in the pathogenesis of ASVD in CKD. Other works have detected IDCs in atherogenic susceptible regions in the aorta of rabbits and mice, in which their abundance was correlated with a genetic susceptibility of mice to ASVD [13]. On the contrary depletion of IDCs was shown to reduce intimal lipid accumulation, indicating that IDCs would be involved in the formation of nascent atherosclerotic lesions [14]. Furthermore, it has been suggested that a proinflammatory environment, such as CKD, might promote IDCs accumulation and predispose these regions to ASVD [13]. The origin of IDCs and the physiological role of IDCs in the normal aorta is still unknown. IDCs isolated from the aorta and valves of mice have the capacity to cross-present antigen to CD8^+^T cells in vitro, but the scarcity of T-cells in the aortic intima suggests that antigen presentation would not be their primary function. In this sense, it has been recently suggested that initial foam cells in early lesions would derive primarily from IDCs rather than from newly recruited monocytes [13].

AIT lesions showed scanty CD40^+^-expressing cells in the intima with their numbers increasing in areas with progressive ASVD lesions as well as in the vessels of the adventitia according to the severity of the lesions (Table 2). In addition, the proportion of CD40L^+^ infiltrating cells was increased in progressive ASVD lesions (Table 2) and CD40L^+^ expression was induced in endothelial cells of vasa vasorum suggesting an endothelial activation. These data are not surprising because the CD40/CD40L system has been already implicated in ASVD pathogenesis [15]. However, no relationship could be established with CKD in this work (Table 3).

**Table 3:** **Relative proportions of infiltrating cells in aorta samples grouped according to presence of CKD and to the modified classification of American Heart Association (AHA)**

			**Non-CKD**				**CKD**		
		**AIT**	**PIT**	**FA**		**AIT**	**PIT**	**FA**	
**Mab**	**Location**	**n = 5**	**n = 5**	**n = 5**	**p**	**n = 5**	**n = 5**	**n = 5**	**p**
αCD3	intima	10 ± 9%	18 ± 10%	25 ± 5%	0.05	14 ± 12%	23 ± 11%	33 ± 6%	0.06
	adventitia	8 ± 5%	11 ± 5%	22 ± 11%	0.14	7 ± 8%	31 ± 31%	23 ± 18%	0.07
αS100	intima	3 ± 1%	3 ± 1%	11 ± 7%	0.11	8 ± 6%	11 ± 8%	16 ± 5%	0.22
	adventitia	10 ± 10%	8 ± 5%	8 ± 3%	0.85	13 ± 9%	9 ± 5%	9 ± 5%	0.78
αCD40	intima	38 ± 19%	70 ± 15%*	84 ± 6%^t^	0.01	30 ± 26%	66 ± 25%	65 ± 12%	0.11
	adventitia	61 ± 10%	69 ± 15%	76 ± 6%	0.16	57 ± 29%	86 ± 7%	66 ± 16%	0.07
αCD40L	intima	28 ± 18%	51 ± 22%	69 ± 31%	0.08	35 ± 14%	70 ± 20%	51 ± 15%	0.06
	adventitia	46 ± 16%	40 ± 15%	53 ± 14%	0.46	58 ± 12%	68 ± 16%	40 ± 9%	0.09
αNF-κB	intima	10 ± 18%	49 ± 30%	46 ± 17%	0.07	19 ± 12%	42 ± 29%	62 ± 32%	0.11
	adventitia	11 ± 11%	58 ± 35%*	31 ± 6%	0.03	14 ± 9%	31 ± 32%	62 ± 18%	0.22

For each case we calculated the number of CD3^+^, S100^+^, CD40^+^, CD40L^+^ and NF-κB^+^ infiltrating cells as percentage of total cells and expressed the result as the mean ± SD.

*P < 0.05 vs AIT (Bonferroni Test); ^t^P < 0.005 vs AIT (Bonferroni test).

Abbreviations:

AIT: adaptive intimal thickening,

PIT: pathological intimal thickening,

FA: fibroatheroma.

CKD: Chronic kidney Disease

**Figure 2:**
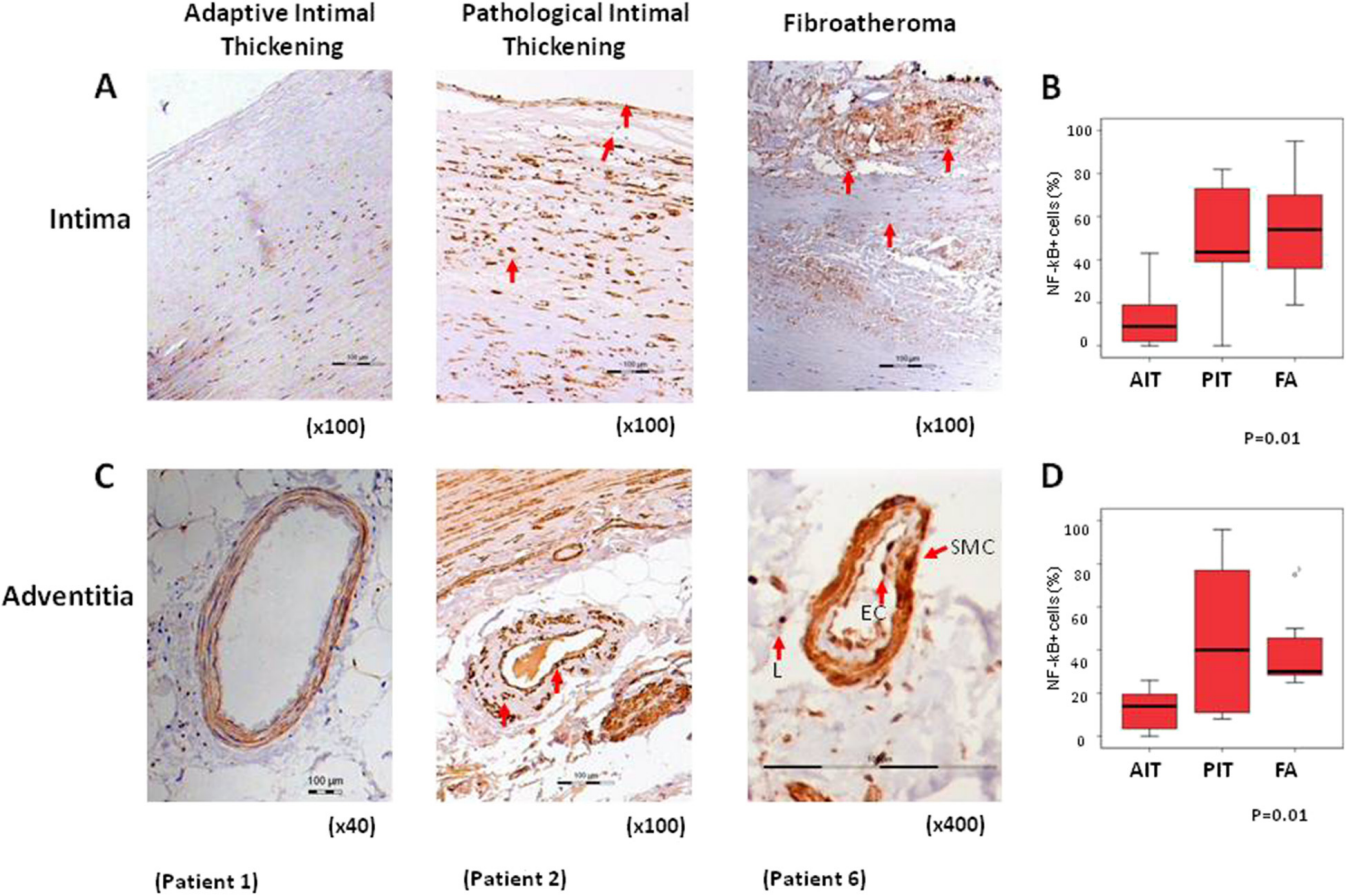
**Increased activation of NF-κB in progressive ASVD lesions. A**. Activated NF-κB-p65 detected in the intima of aortas with PIT or FA by immunostaining with anti phospho S536. Sample with AIT did not show activation of NF-κB Magnifications as indicated. **B**. Box-plot showing percentage of cells with activated NF-κB in the intima, regarding the total number of cells in the field, according to histological lesion. Kruskal-Wallis test. **C**.- Activated NF-κB-p65 detected in the adventitia of aortas with PIT or FA by immunostaining with anti-phospho S536. Activation of NF-κB was detected in endothelial cells (ECs), smooth muscle cells (SMCs) and lymphocytes (L). Sample with AIT did not show activation of NF-κB. Magnifications as indicated. **D**.- Box-plot showing percentage of cells with activated NF-κB in the adventitia, regarding the total number of cells in the field, according to histological lesion. Kruskal-Wallis test.

Since NF-κB is a downstream target of CD40/CD40L signalling we analysed NF-κB activation in the progressive ASVD lesions, as well as its association with the accumulation of inflammatory cells, by using an antibody specific for the phospho-S536 form of NF-κB-p65 (see Methods). Activation of NF-κB was detected in endothelial cells (ECs), smooth muscle cells (SMCs) and lymphocytes (Figure 2A, second and third panels). Furthermore, numbers of activated NF-κB^+^ cells in the intima correlated positively with the severity of ASVD lesions (r = 0.56, p = 0.003) (Figure 2), and with the percentage of CD3^+^ cells in the adventitia (r = 0.50, p = 0.008), of CD40^+^ cells (r = 0.59, p = 0.002 in the intima, and r = 0.45, p = 0.02 in the adventitia), and of CD40L^+^ cells (r = 0.44, p = 0.03 in the intima). No differences were detected in the number of activated NF-κB^+^ cells in aortas from CKD patients vs. controls without CKD.

In summary, our study showed an accumulation of infiltrated CD3^+^ lymphocytes, of CD40^+^ cells as well as an increase in the activation of NF-κB associated to progressive ASVD lesions in the abdominal aorta. The arterial wall of abdominal aorta displayed an increase number of intimal dendritic cells (IDCs) in patients with CKD suggesting a role of these cells in the pathogenesis of ASVD in CKD.

## Abbreviations

ASVD: Atherosclerotic Vascular Disease
CKD: Chronic Kidney Disease
AIT: Adaptive Intimal Thickening
PIT: Pathological Intimal Thickening
FA: Fibroatheroma
IDCs: Intimal Dendritic Cells
aNF-κB: Activated Nuclear Factor-κB
